# Beneficial effects of brief early life angiotensin-converting enzyme inhibition wane with time in sheep with solitary functioning kidney

**DOI:** 10.1042/CS20220811

**Published:** 2023-04-19

**Authors:** Zoe McArdle, Reetu R. Singh, Helle Bielefeldt-Ohmann, Karen M. Moritz, Kate M. Denton, Michiel F. Schreuder

**Affiliations:** 1 Cardiovascular Program, Monash Biomedicine Discovery Institute and Department of Physiology, Monash University, Melbourne, Australia; 2 School of Chemistry & Molecular Biosciences, The University of Queensland, St Lucia, Queensland, Australia; 3 Child Health Research Centre and School of Biomedical Sciences, The University of Queensland, Brisbane, Queensland, Australia; 4 Department of Pediatric Nephrology, Amalia Children’s Hospital, Radboud University Medical Center, Nijmegen, The Netherlands

**Keywords:** angiotensin converting enzyme inhibition, hyperfiltration, kidney disease, renal functional reserve, Solitary functioning kidney

## Abstract

A child with a congenital solitary functioning kidney (SFK) may develop kidney disease from early in life due to hyperfiltration injury. Previously, we showed in a sheep model of SFK that brief angiotensin-converting enzyme inhibition (ACEi) early in life is reno-protective and increases renal functional reserve (RFR) at 8 months of age. Here we investigated the long-term effects of brief early ACEi in SFK sheep out to 20 months of age. At 100 days gestation (term = 150 days) SFK was induced by fetal unilateral nephrectomy, or sham surgery was performed (controls). SFK lambs received enalapril (SFK+ACEi; 0.5 mg/kg, once daily, orally) or vehicle (SFK) from 4 to 8 weeks of age. At 8, 14 and 20 months of age urinary albumin excretion was measured. At 20 months of age, we examined basal kidney function and RFR via infusion of combined amino acid and dopamine (AA+D). SFK+ACEi resulted in lower albuminuria (∼40%) at 8 months, but not at 14 or 20 months of age compared with vehicle-SFK. At 20 months, basal GFR (∼13%) was lower in SFK+ACEi compared with SFK, but renal blood flow (RBF), renal vascular resistance (RVR) and filtration fraction were similar to SFK. During AA+D, the increase in GFR was similar in SFK+ACEi and SFK animals, but the increase in RBF was greater (∼46%) in SFK+ACEi than SFK animals. Brief ACEi in SFK delayed kidney disease in the short-term but these effects were not sustained long-term.

## Introduction

A congenital solitary functioning kidney (SFK) predisposes children to hypertension, albuminuria and kidney disease early in life [1,2]. Indeed, ∼26% of children with a SFK have hypertension by ∼5 years of age and ∼19% are diagnosed with proteinuria by ∼10 years of age [1]. In response to a reduction in kidney mass, glomerular hypertrophy and hyperfiltration initially normalize kidney function but over time may contribute to the pathophysiology of kidney disease [3].

Renal functional reserve (RFR) is the increase in glomerular filtration rate (GFR) from baseline induced by a vasodilatory stimulus (protein load, intravenous amino acid and/or dopamine infusion) [4]. A reduced RFR can occur in states of low nephron number with glomerular hyperfiltration despite GFR remaining within normal ranges [5]. Impaired RFR has been observed in hypertension [6], adult kidney donors [7], progressive kidney disease [8] and children with SFK [5].

Angiotensin converting enzyme inhibitors (ACEi) are efficient in reducing blood pressure (BP), proteinuria and risk of kidney disease progression in children with chronic kidney disease (CKD) [9,10]. In addition, ACEi at a critical window early in life has been shown to prevent the development of hypertension and improve kidney function in spontaneously hypertensive rats (SHR), a benefit that persists up to ∼16 months after treatment withdrawal [11,12]. We have recently shown that brief ACEi treatment from 4 to 8 weeks of age in an ovine model of SFK, delayed kidney hypertrophy and prevented albuminuria independent of BP lowering, 6 months after treatment withdrawal [13]. In addition, we found mitigation of glomerular hyperfiltration (lower GFR and filtration fraction), enhanced RFR and increased nitric oxide (NO) bioavailability [13], factors that are impaired in SFK sheep [14–16]. Taken together, these data indicate that modifying glomerular haemodynamics by early life ACEi in a sheep model of congenital SFK, with similar kidney development and maturation as humans, may preserve kidney lifespan. Therefore, in the present study, we followed a separate cohort of animals to 20 months of age to determine if the reno-protection persisted.

In the present study we examined whether ACEi (enalapril) from 4 to 8 weeks in SFK lambs (1) reduced albuminuria beyond 8 months of age, (2) reduced hyperfiltration (lower GFR and filtration fraction) at 20 months of age, (3) increased vasodilation of renal vasculature at 20 months of age, or (4) increased the RFR response to a combined infusion of amino acids and dopamine at 20 months of age, 18 months after treatment cessation.

## Materials and methods

### Animals

An Animal Ethics Committee of Monash University approved experimental procedures (Ethics numbers: MARP/182/2016, #20442), which were performed in accordance with the guidelines of the National Health and Medical Research Council of Australia. All sheep were housed at the Monash University's Gippsland Field Station in between surgical/experimental procedures, and transported to Monash Animal Research Platform for surgical/experimental procedures. Surgical procedures were performed under isoflurane anesthesia, with a detailed description of the surgical and experimental procedures previously published [13]. Briefly, a congenital SFK was induced by unilateral nephrectomy in the sheep fetus on the 100th day of a 150-day gestation (SFK; *n*=19). A sham procedure was also performed (sham; *n*=9). Only male fetuses were used in the present study. SFK lambs aged 4 weeks were randomly assigned to undergo ACEi via oral administration of enalapril (maleate salt, E6888, 0.5 mg/kg/day; *n*=10; SFK+ACEi group) or vehicle (water; *n*=9; SFK group). At 6 months of age, lambs underwent surgery to construct carotid artery loops, allowing direct access to the carotid artery for catherization to measure blood pressure (BP) and collect blood samples. At 20 months of age sheep underwent surgery under general anesthesia for insertion of a bladder catheter as previously detailed [17] for the measurement of kidney function.

### Kidney volume, basal cardiovascular and kidney function

At 2, 6 and 20 months of age kidney volume was determined by magnetic resonance imaging (MRI), via a three-dimensional T1 VIBE DIXON sequence with a Siemens Skyra (Siemens, Erlangen, Germany), as previously described [13]. At 8 and 14 months of age, sheep underwent a body fluid balance study where food and water intake and urine output were monitored, and 24-h urinary albumin excretion was determined over 5 days. At 8 and 14 months of age, BP (systolic, diastolic, mean) and heart rate (HR) were measured via an indwelling arterial catheter over a 72-h period. At 20 months of age, following surgical insertion of a bladder catheter and recovery, basal GFR and renal blood flow (RBF) were measured via ^51^Chromium ethylenediaminetetraacetic acid (^51^Cr EDTA) and para-aminohippuric acid (PAH) clearance, respectively, over a 7-h period with BP and HR measured continuously during this period. A 1-h urine sample collected in this basal period was used to determine albumin excretion (expressed as mg/24 h) at this age.

### Cardiovascular and kidney function in response to amino acid and dopamine infusion

Response to combined amino acid and dopamine (AA+D) infusion was determined 2 days after basal cardiovascular and kidney function measurement. To establish baseline BP, HR and kidney function were measured for 60 min following one hour of equilibration. Then intravenous infusion of amino acids (0.065 ml/kg/hour of a 10% solution without electrolytes, Synthamin 17®, AHA692, Baxter) plus dopamine (5 μg/kg/min, dopamine hydrochloride, H8502, Sigma-Aldrich) was administered for 2 h. Within 2–3 days of experimentation, sheep were euthanized with an overdose of Pentobarbitone sodium (100 mg/kg, iv.).

### Sample analysis

^51^Cr EDTA levels were measured via a gamma counter (PerkinElmer Wizard 1470). PAH concentration was determined using a previously described rapid microplate assay [18]. Plasma renin activity (PRA) was determined by radioimmunoassay (ProSearch International Pty, Malvern, Australia) from blood withdrawn via jugular vein puncture in lambs (4–8 weeks of age), which in the ACEi treated animals occurred approximately 1 h after treatment at each age, and arterial blood samples collected at 8, 14 and 20 months of age. Urinary albumin levels were assayed via an Albuwell O-ovine microalbuminuria ELISA kit (Exocell, 1013, Philadelphia, PA) as per manufacturer’s instructions. Urinary protein concentration was determined using the Pierce™ Coomassie (Bradford) protein assay kit (23236, ThermoFisher Scientific) and corrected for urine flow. Renal vascular resistance (RVR) was calculated as (MAP/RBF), and filtration fraction was calculated as (GFR/effective renal plasma flow). Urinary sodium excretion (U_NA_V) was measured (Beckman Coulter, Monash Medical Centre) and corrected for urine flow. Filtered load sodium was determined as (plasma sodium concentration × GFR), and fractional sodium excretion (FE_NA_%) calculated as ([U_NA_V/filtered load sodium] × 100).

### Tissue analysis

Kidneys were immersion fixed in 4% paraformaldehyde and paraffin embedded for histological analysis (Monash histology platform). Sections were blinded and assessment of fibrosis, glomerular diameter and kidney histopathology (assessed by expert pathologist H.B-O), were performed via Masson’s trichrome and Periodic acid–Schiff stains. Kidney histopathology was scored on the basis of pathology severity, assigned as follows: zero, no lesions apparent; one, minimal change; two, mild change; three, moderate change; four, marked/severe change(s) present.

### Statistical analysis

All values are presented as mean ± SEM. Statistical analysis was performed using GraphPad Prism 9.0 (GraphPad software Inc., CA, U.S.A.), statistical significance was accepted as *P*≤0.05. Data were tested for normality using a Shapiro–Wilk test and for data that violated normality a rank-based test was performed. An analysis of variance was performed (body weight, kidney volume, MAP, HR, albumin) by fitting a mixed model ANOVA examining the effects of two factors: group (*P*_group_; sham, SFK, or SFK+ACEi), age (*P*_age_) and their interaction (*P*_group×age_). A two-way ANOVA was performed examining the effects of group (*P*_group_; sham, SFK or SFK+ACEi) and AA+D (*P*_AA+D_; basal and during AA+D infusion) and their interaction. Basal kidney function variables, absolute change from baseline in response to AA+D, and kidney histopathology were analyzed by a one-way ANOVA, and where appropriate a Dunnett’s post-hoc analysis was performed (comparing with SFK group).

## Results

### Plasma renin activity (PRA) early in life

**Figure 1:**
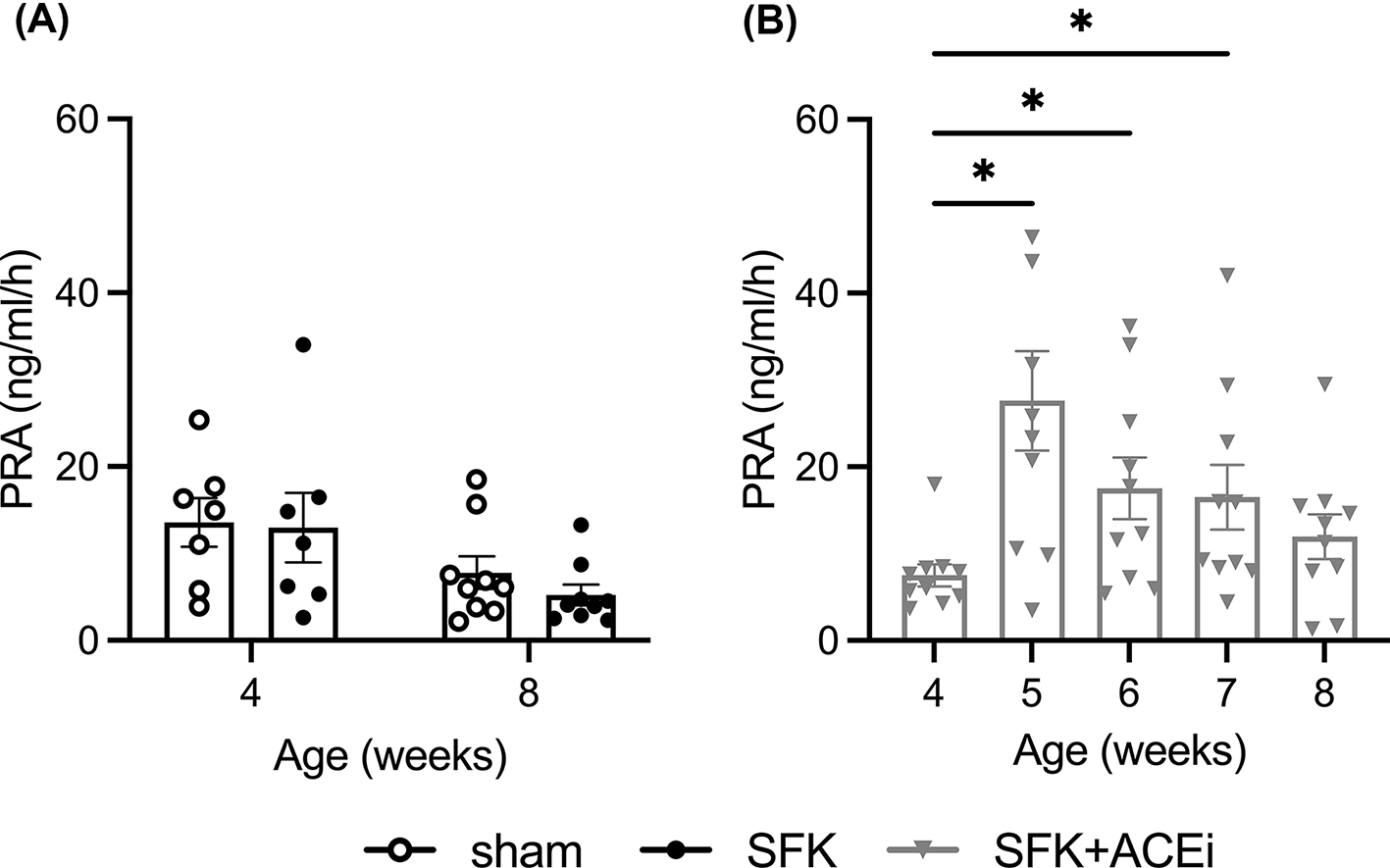
During treatment in SFK sheep plasma renin activity (PRA) increases, an index of effective ACE inhibition (**A**) PRA at 4 and 8 weeks of age in lambs that underwent fetal sham surgery (*n*=7–9) or fetal uninephrectomy (SFK; *n*=7–9). (**B**) PRA at 4, 5, 6, 7 and 8 weeks of age in male lambs that underwent fetal uninephrectomy prior to (4 weeks) and during administration of enalapril for 4 weeks (SFK + ACEi; *n*=10). Data represented as mean ± S.E.M. *P* values are from (A) a two-way mixed effect ANOVA, followed by a Bonferroni post-hoc comparing between sham and SFK groups at 4 and 8 weeks and (B) repeated measures one-way ANOVA followed by a Dunnett's post-hoc, *P<0.05 compared with 4 weeks in SFK + ACEi animals.

PRA was similar between sham and SFK sheep at 4 and 8 weeks of age (Figure 1A). In SFK sheep, ACEi treatment significantly increased PRA (Figure 1B). Compared with pre-ACEi levels at 4 weeks of age, PRA was ∼267% greater at 5 weeks, ∼133% greater at 6 weeks, and ∼120% greater at 7 weeks but was not significantly different at 8 weeks of age (Figure 1B).

### Body weights and kidney volume at 2, 6 and 20 months of age

**Figure 2:**
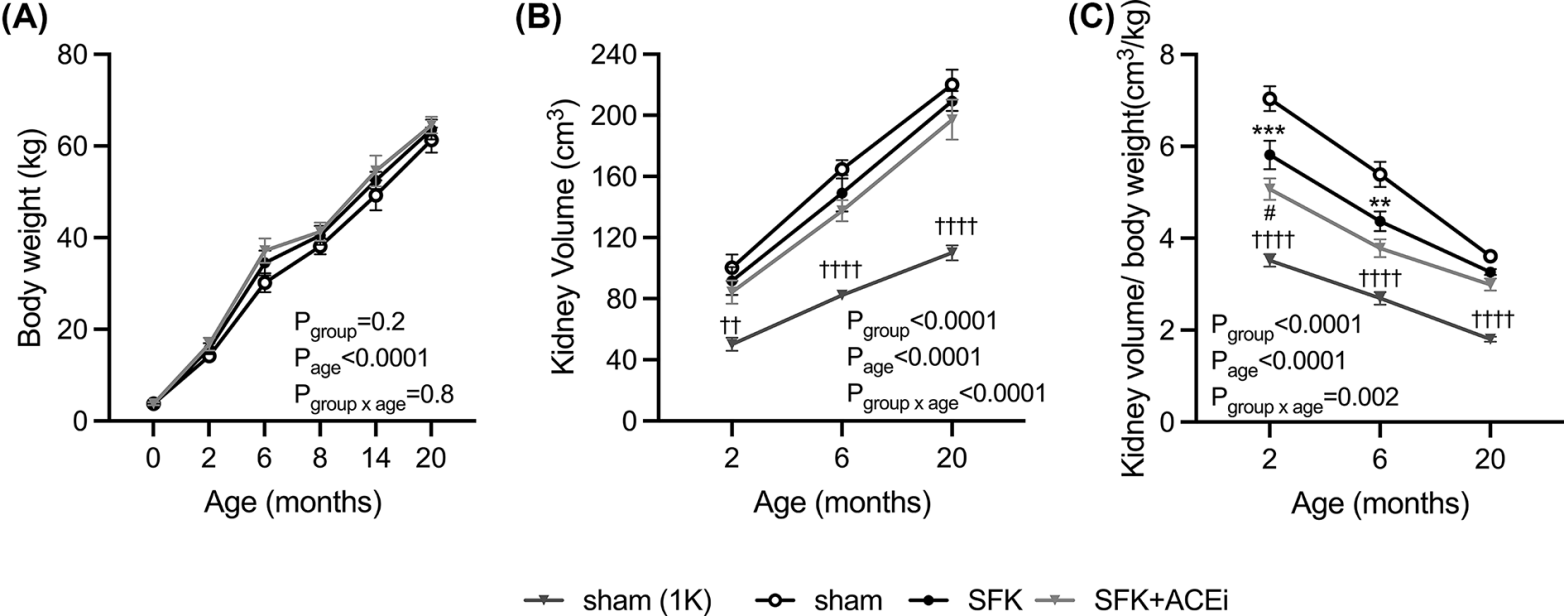
Brief early ACEi treatment in SFK sheep reduces kidney volume at 2 months (end of treatment period) (**A**) Body weight from birth (0 mth) to 20 months, (**B**) Kidney volume (cm^3^) and (**C**) Kidney volume normalized to body weight (cm^3^/kg) at 2, 6 and 20 months of age. Data presented in male lambs that underwent fetal sham surgery (sham; *n*=9), fetal uninephrectomy (SFK; *n*=9) or fetal uninephrectomy and angiotensin-converting enzyme inhibition via enalapril between 4 and 8 weeks of age (SFK+ACEi; *n*=10). Data in graphs (B and C) also depict one kidney values for sham animals (sham (1K)). Data are mean ± S.E.M. *P* values are from Dunnett’s post-hoc test following a two-way repeated measures ANOVA. **P<0.01, ***P<0.001 comparing between sham vs. SFK and #*P*<0.05 comparing between SFK vs. SFK+ACEi groups, †† *P*<0.01, ††††*P*<0.0001 comparing between sham (IK) vs. SFK.

Birth weight and body weights during the study were similar between groups (Figure 2A). Total kidney volume, presented as one or two kidneys for sham sheep and one kidney for SFK and SFK+ACEi sheep, increased with age in all groups (*P*_age_<0.0001, Figure 2B). Total kidney volume was significantly greater in SFK sheep (∼45–47%) than a single kidney of a sham and similar between sham (two kidneys), SFK and SFK+ACEi groups at 2, 6 and 20 months of age (Figure 2B). Total kidney volume normalized to body weight decreased with age in all groups (*P*_age_<0.0001, Figure 2C). At 2 months of age kidney volume normalized to body weight was ∼17% lower in the SFK group compared with sham (two kidney) counterparts and ∼40% greater compared with sham (one kidney) counterpart (both *P*<0.0001, Figure 2C). At the end of ACEi treatment (2 months of age) SFK+ACEi animals had a ∼13% lower normalized kidney volume compared with SFK animals (*P*=0.02, Figure 2C). At 6 months of age kidney volume normalized to body weight was ∼19% lower in SFK animals compared with sham (two kidneys) (*P*=0.001, Figure 2C) and ∼38% greater than sham (one kidney) (*P*<0.0001, Figure 2C), but similar between SFK and SFK+ACEi animals. At 20 months of age normalized kidney volume was ∼45% greater in SFK sheep compared with sham (one kidney) counterparts (*P*<0.0001) and similar between sham (two kidneys), SFK and SFK+ACEi groups (Figure 2B).

### Basal mean arterial pressure (MAP), heart rate (HR), urinary albumin excretion and PRA at 8, 14 and 20 months of age

MAP was significantly higher in SFK sheep at all ages compared with sham (*P*_group_<0.0001, Table 1). However, MAP was similar in SFK and SFK+ACEi groups at each age (Table 1). HR decreased with age (*P*_age_<0.0001, Table 1), but was similar between groups at each age. Urinary albumin excretion was significantly greater in SFK (∼47–155%) compared with sham animals at all ages (Table 1). At 8 months of age, urinary albumin was significantly lower (∼40%) in SFK+ACEi sheep compared with SFK (*P*=0.04, Table 1). However, urinary albumin excretion was not different between SFK+ACEi and SFK groups at 14 and 20 months of age (Table 1). Urine flow was similar between groups at all ages (Table 1). PRA declined with age in all groups (*P*_age_<0.0001, Table 1) and was significantly lower in SFK sheep compared with sham sheep at all ages (*P*_group_<0.008, Table 1). PRA was similar between SFK+ACEi and SFK animals at all ages.

**Table 1:** Basal cardiovascular and renal parameters in sham (*n*=9), SFK (*n*=9) and SFK+ACEi (*n*=10) sheep

Variable	Age	Sham	SFK	SFK+ACEi
MAP (mm Hg)	8 months	82 ± 2	91 ± 1**	97 ± 2
	14 months	83 ± 2	93 ± 2***	97 ± 2
	20 months	81 ± 1	97 ± 2****	93 ± 1
HR (beats/min)	8 months	101 ± 6	94 ± 4	91 ± 5
	14 months	80 ± 2	78 ± 2	80 ± 2
	20 months	81 ± 1	78 ± 1	79 ± 1
Albumin excretion (mg/24 h)	8 months	32.0 ± 8.8	60.6 ± 6.3*	36.5 ± 3.9#
	14 months	48.4 ± 7.6	124.0 ± 12.3***	91.2 ± 19.0
	20 months	160.1 ± 29.2	362.3 ± 70.2*	295.2 ± 54.0
UF (ml/min/kg)	8 months	0.02 ± 0.007	0.02 ± 0.006	0.02 ± 0.002
	14 months	0.03 ± 0.006	0.02 ± 0.004	0.02 ± 0.004
	20 months	0.02 ± 0.003	0.02 ± 0.003	0.02 ± 0.003
PRA (ng/ml/h)	8 months	4.7 ± 0.4	2.9 ± 0.3*	3.1 ± 0.6
	14 months	2.1 ± 0.2	0.8 ± 0.2***	2.0 ± 0.6
	20 months	1.4 ± 0.2	0.8 ± 0.1*	1.2 ± 0.3

Data are presented as mean ± SEM. *P<0.05, **P<0.01, ***P<0.001, ****P<0.0001 comparing between sham and SFK groups, #*P*<0.05 comparing between SFK and SFK+ACEi groups analyzed via two-way mixed model ANOVA followed by post-hoc Dunnett's test. Mean arterial pressure (MAP), heart rate (HR), urine flow (UF) and plasma renin activity (PRA). Note for albumin, urine flow and PRA data 8 months of age represents *n*=8, sham; *n*=8, SFK; SFK+ACEi *n*=9; for MAP and HR data 8 months of age represents *n*=8, sham; *n*=7, SFK; SFK+ACEi *n*=9.

### Basal kidney function at 20 months of age

**Figure 3:**
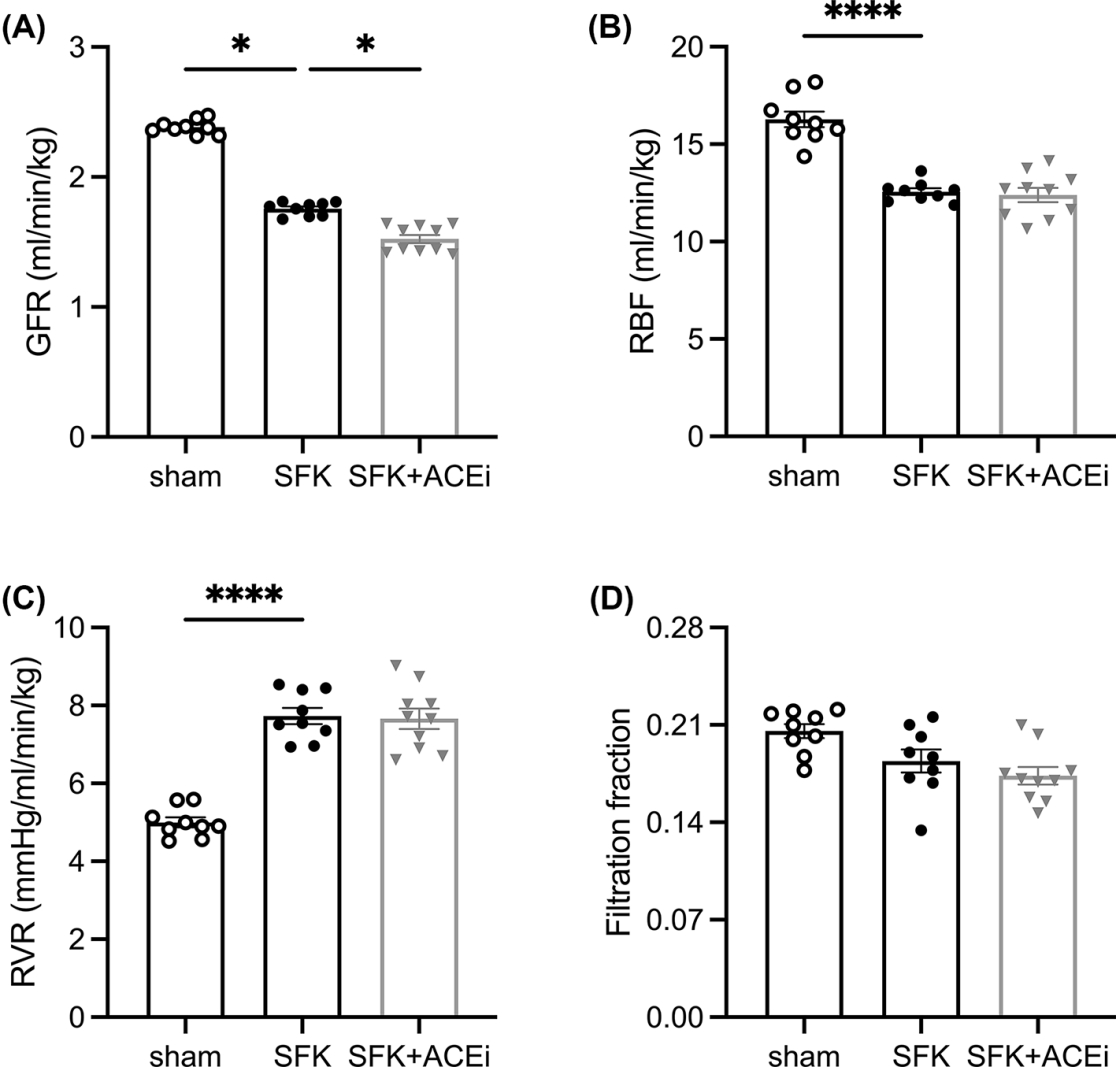
Brief early ACEi treatment in SFK sheep reduces GFR at 20 months of age Basal kidney hemodynamics (**A**) glomerular filtration rate (GFR) (**B**) renal blood flow (RBF) (**C**) renal vascular resistance (RVR), (**D**) filtration fraction measured over 7 h presented as a singular average of this period at 20 months. Data presented in male lambs that underwent fetal sham surgery (sham; *n*=9), fetal uninephrectomy (SFK; *n*=9) or fetal uninephrectomy and angiotensin-converting enzyme inhibition via enalapril between 4 and 8 weeks of age (SFK+ACEi;* n*=10). Data are mean ± S.E.M. *P* values are from Dunnett’s post-hoc test following one-way analysis of variance for parametric data (B–D) and from Dunn’s post-hoc test following a Kruskal–Wallis test for non­parametric data (A). *P<0.05, ****P<0.0001 comparing between sham vs. SFK and SFK vs. SFK+ACEi groups.

At 20 months of age, SFK sheep had a significantly lower GFR (∼26%, *P*=0.04, Figure 3A) and RBF (∼23%, *P*<0.0001, Figure 3B) and greater renal vascular resistance (RVR) (∼55%, *P*<0.0001, Figure 3C). Filtration fraction was similar between SFK and sham sheep (Figure 3D). Compared with SFK sheep, SFK+ACEi sheep had a significantly lower GFR (∼13%, *P*=0.02, Figure 3A). However, RBF, RVR and filtration fraction were not different between SFK and SFK+ACEi sheep (Figure 3B–D).

### Cardiovascular and kidney function in response to combined AA+D infusion at 20 months of age

**Figure 4:**
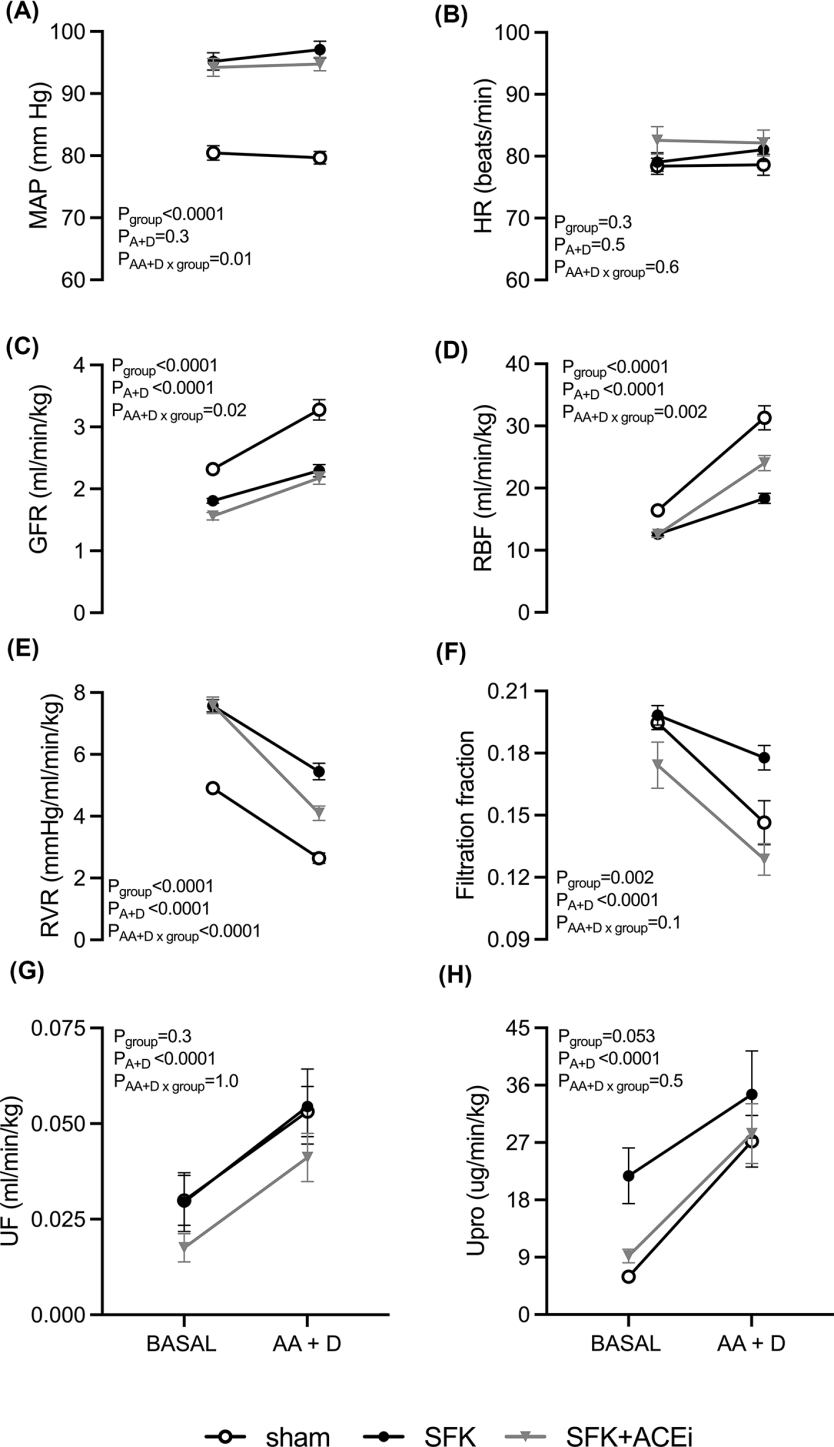
Brief early ACEi treatment in SFK sheep increases the kidney vasodilatory response to AA+D infusion but not RFR at 20 months of age Variables were measured before and during amino acid and dopamine infusion (AA+D) in conscious sheep (sham; *n*=9, SFK; *n*=9, SFK+ACEi; *n*=10). Renal variables are presented as absolute values corrected for body weight. (**A**) Mean arterial pressure (MAP), (**B**) heart rate (HR), (**C**) glomerular filtration rate (GFR) (**D**) renal blood flow (RBF), (**E**) renal vascular resistance (RVR), (**F**) filtration fraction, (**G**) urine flow (UF) and (**H**) urinary protein excretion (Upro). Data are mean ± S.E.M. *P* values represent the results from repeated-measure s ANOVA, with factors group (sham, SFK, SFK+ACEi), AA+D (amino acid dopamine infusion), and their interaction.

AA+D infusion had no significant effect on MAP or HR in any group (Figure 4A,B). AA+D infusion caused RVR to decrease from baseline, which resulted in an increase in RBF and GFR in all groups (all *P*_AA+D_<0.0001, Figure 4C–E). Given the increase in RBF was greater than the increase in GFR, FF fell from baseline in all groups (*P*_AA+D_<0.0001, Figure 4F). In response to AA+D infusion the magnitude of the decrease in RVR and FF were similar between SFK and sham animals (Figure 4E,F). In the SFK group compared with sham animals, the increase in GFR (∼0.5 ml/min/kg less than sham, *P*=0.01, Figure 4C) and RBF (∼9.2 ml/min/kg less,* P*=0.0001, Figure 4D) were less in response to AA+D infusion. AA+D infusion caused a greater decrease in RVR (∼1.4 mmHg/ml/min/kg, *P*<0.0001, Figure 4E) and a greater increase in RBF (∼5.8 ml/min/kg, *P*=0.008, Figure 4D) in SFK+ACEi animals compared with SFK. However, the increment in GFR and reduction in FF were similar between SFK+ACEi and SFK animals (Figure 4C,F)

### Kidney excretory function in response to combined AA+D infusion at 20 months of age

**Figure 5:**
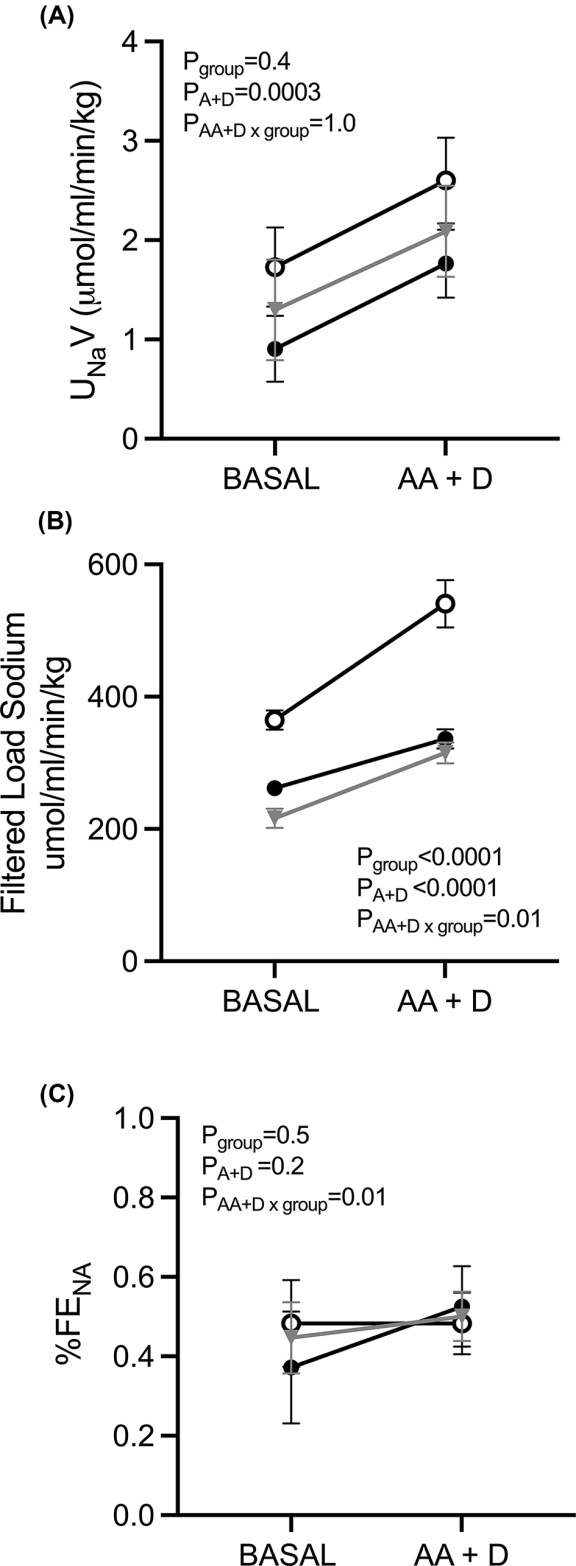
Brief early ACEi treatment in SFK sheep has no effect on urinary sodium excretion and filtered load sodium in response to AA+D infusion at 20 months of age Variables were measured before and during amino acid and dopamine infusion (AA+D) in conscious sheep (sham; *n*=9, SFK; *n*=9, SFK+ACEi; *n*=10). Excretory variables are presented as absolute values corrected for body weight. (**A**) urinary sodium excretion (U_na_V), (**B**) filtered load sodium, (**C**) fractional sodium excretion (FE_NA_%). Data are mean ± S.E.M. *P* values represent the results from repeated-measures ANOVA, with factors group (sham, SFK, SFK+ACEi), AA+D (amino acid dopamine infusion), and their interaction.

Urine flow, urinary protein excretion (both *P*_AA+D_<0.0001, Figure 4G,H), urinary sodium excretion (*P*_AA+D_ = 0.0003, Figure 5A) and filtered load sodium (*P*_AA+D_<0.0001, Figure 5B) increased from baseline in all groups in response to AA+D infusion. AA+D infusion had no significant effect on fractional sodium excretion (Figure 5C). The magnitude of the increase in urine flow, urinary protein excretion and urinary sodium excretion were similar between groups. The magnitude of the increase in filtered load sodium was significantly less (∼102 μmol/ml/min, *P*=0.02) in SFK compared with sham animals and similar between SFK and SFK+ACEi animals (Figure 5B)

### Post-mortem and kidney histopathology at 20 months of age

Body weight, kidney weight and kidney weight normalized to body weight were similar between groups (Table 2). Heart weight and heart weight normalized to body weight were significantly greater in SFK compared with sham animals, but similar between SFK and SFK+ACEi animals (Table 2). Mean glomerular diameter was significantly greater in SFK sheep compared with sham, an index of glomerular hypertrophy, but was similar between SFK+ACEi and SFK animals (Table 2). Percentage of cortical fibrosis was similar between groups. The overall pathology score, determined by an expert veterinary pathologist, was significantly greater in SFK compared with sham and similar between SFK+ACEi and SFK animals (Table 2).

**Table 2:** Post-mortem and kidney histopathology data in sham, SFK and SFK+ACEi sheep at 20 months of age

Variable	Sham (*n*=9)	SFK (*n*=9)	SFK+ACEi (*n*=10)
Body weight (kg)	61 ± 3	64 ± 2	64 ± 2
Total kidney weight (g)	143 ± 7	130 ± 8	121 ± 4
Kidney weight per body weight (g/kg)	2.4 ± 0.2	2.0 ± 0.1	1.9 ± 0.1
Heart weight (g)	161 ± 5	196 ± 6**	200 ± 8
Heart weight per body weight (g/kg)	2.7 ± 0.1	3.1 ± 0.1*	3.1 ± 0.1
Mean glomerular diameter (μm)	110 ± 2	126 ± 2***	127 ± 3
Cortical fibrosis (%)	5.6 ± 0.5	4.8 ± 0.8	5.2 ± 0.6
Pathology score	0.4 ± 0.2	1.4 ± 0.3*	1.5 ± 0.3

Data are presented as mean ± SEM. *P<0.05, ** *P*<0.01, ***P<0.001, comparing between sham and SFK groups analyzed via one-way ANOVA followed by Dunnett’s post-hoc test.

## Discussion

The main finding of the present study was that a brief period of ACEi in early life reduced albuminuria in sheep born with a SFK at 8 months of age, confirming our earlier report [13], but this effect waned over time. However, residual differences in kidney function in adult SFK sheep remained 18 months following cessation of ACEi treatment. This included greater renal vasodilation in response to an AA+D infusion, though unlike our early study at 8 months of age, this was not sufficient to cause a greater increase in GFR. Significantly, at 20 months of age, GFR was lowered to a similar extent in the SFK+ACEi group as observed at 8 months of age [12]. Together these data indicate that brief early life ACEi has altered the trajectory of kidney disease in SFK, but future studies examining modification of the window and duration of ACEi treatment are required to establish if outcomes may be improved long-term.

In the present study, the prevention of albuminuria observed at 8 months of age in SFK sheep treated briefly with ACEi early in life was not sustained in the long-term with elevations in urinary albumin excretion having returned to SFK levels at 14 and 20 months of age. The degree to which albuminuria was reduced at 8 months of age mirrored our previous findings in a separate cohort of sheep [13]. Interestingly, in the ESCAPE trial in children with CKD and hypertension who received long-term ACEi, an early anti-proteinuric effect was followed by a gradual increase in proteinuria to pre-treatment levels after 3 years of follow-up despite blood pressure control [9]. In children with hypodysplastic CKD, it has been shown that continuous ACEi reduced blood pressure, but did not slow kidney functional decline. This may indicate ACEi in established CKD may not be effective in altering its course [19]. In the present study, brief early life ACEi in SFK did not prevent the elevation in blood pressure at 8, 14 or 20 months of age. Given blood pressure control is an important target to slow progression of kidney disease in children [9], the persistent elevation in blood pressure in SFK+ACEi animals may have contributed, at least in part, to the rebound in albuminuria observed by 14 months of age. In the Prague hypertensive rat, the long-term blood pressure lowering effects of brief treatment with losartan, from 5 to 9 weeks of age, were intensified by a second treatment window between 15 and 19 weeks of age [20]. Thus, modulation of treatment dose, window or regime in SFK sheep may prolong the reno-protective benefits and/or result in blood pressure lowering effects and, this needs to be examined in future studies.

In the present study, early life ACEi in SFK sheep resulted in ∼13% lower basal GFR than SFK sheep 18 months after withdrawal of ACEi. A similar reduction in GFR was seen between the groups at 8 months of age in our earlier study [13], which may suggest that CKD has not progressed over this period in either group. In our previous study, a lower GFR and a higher RBF resulted in a lower filtration fraction in SFK+ACEi compared with SFK sheep at 8 months of age [13]. This was accompanied by a lower albumin excretion in SFK+ACEi than SFK sheep, indicating a reduction in hyperfiltration-mediated injury. However, in the present study at 20 months of age, SFK+ACEi sheep had lower GFR than SFK but RBF, FF and albuminuria were similar between the groups. Taken together this suggests that the beneficial effects of early life ACEi in SFK on kidney hemodynamics wanes over time. This lower GFR at 20 months of age could be due to a reduction in ultrafiltration pressure, which would suggest differential regulation of renal segmental vascular resistances between the SFK and SFK+ACEi groups. Alternatively, the lower GFR in SFK+ACEi animals could be due to a reduction in glomerular ultrafiltration coefficient due to reduced hydraulic conductivity and/or reduced filtration surface area (FSA) [21,22]. However, a reduction in FSA seems unlikely given glomerular diameter and kidney volume (measured by MRI) were similar between SFK and SFK+ACEi animals. This sheep SFK model is associated with compensatory nephrogenesis, at 130 days of gestation nephron number was ∼45% more in the SFK compared with a single kidney of a sham sheep and nephrogenesis complete by birth as in humans [23–25]. This indicates that the increase in kidney volume in SFK and SFK+ACEI sheep is partly driven by an increase in nephron number and hypertrophy. There is variation in filtration fraction in the SFK+ACEi animals at 20 months of age, which may indicate that the response to brief early ACEi is waning at variable rates within the group. This is consistent with human studies where there is inter-individual variation in the protein lowering response to ACEi [26]. Whether this lower GFR observed in SFK+ACEi animals delays or accelerates the loss of kidney function compared to SFK sheep requires further investigation beyond 20 months of age. Collectively these observations indicate that that the reno-protective effects of this regimen of brief ACEi early in life are not sustained long-term in SFK.

In the present study, in response to AA+D the increase in GFR was similar between the SFK and SFK+ACEi groups, but SFK+ACEi sheep had a greater decrease in RVR and increase in RBF than SFK. It is possible that residual structural remodelling of the glomerular resistance vessels in the kidney by early life ACEi in SFK increased the capacity for vasodilation driving this response to AA+D [27]. The greater reduction in RVR in response to recruitment of functional reserve in the SFK+ACEi animals compared with SFK, may indicate that these animals maybe more susceptible to glomerular damage due to an increase in glomerular pressure during a physiological challenge. NO also plays an important role in the vasodilatory response to amino acid and/or dopamine infusion [28,29]. In this model of SFK, renal NO bioavailability, determined by total urinary nitrate and nitrite (NOx) excretion, is lower than in sham animals [14,30]. However, previous evidence suggests that urinary NOx excretion does not reflect acute changes in systemic and/or renal NO in response to physiological challenges that alter NO production [31]. Therefore, while not measured in the present study, it is possible that SFK+ACEi sheep have a greater capacity to generate NO in response to AA+D infusion, resulting in the greater increment in RBF compared with SFK sheep.

Systemic infusion of dopamine, at low doses, causes renal vasodilation via D1 [32] and D2-like receptors [33]. Stimulation of D1 receptors increases RBF without affecting GFR in the adult Wistar Kyoto rat but this vasodilation is blunted in the SHR counterpart [34]. However, pretreatment with an AT1R blocker, restores the RBF response and also increases GFR in SHR in response to D1 receptor stimulation [34]. In SHR and rats subjected to subtotal nephrectomy renal D1R dysfunction associated with receptor G-protein uncoupling occurs [35,36]. ATIR and D1R interact forming a heteromeric-signaling complex in the kidney with stimulation of one receptor preventing the signaling of the other [37]. Therefore, by reducing AT1R stimulation with ACEi during early life in SFK it is possible that D1R signaling was improved and still present 18 months after treatment withdrawal driving the greater renal vasodilatory response to AA+D. Future studies need to examine kidney hemodynamic responses to D1 receptor stimulation in SFK sheep and in response to ACEi in SFK to confirm this.

Strengths of this study include the similarity in timing of nephrogenesis in sheep and humans and that functional measurements were conducted in conscious sheep. Additionally, the dose of enalapril (0.05 mg/kg/day) used in the current study is similar to clinical doses of ACEi used in children with CKD [38]. Limitations of the present study include lack of blood pressure and renal function measurement at the end of the treatment window or any time before 8 months of age. ACEi likely decreased BP during the treatment window, as this has been reported previously in lambs [39], but it would be of interest to know if, and for how long, BP remained low after cessation of treatment. Serial measurement of kidney function could not be performed because of the difficulty of placing bladder catheters in male sheep. Thus, GFR measurements were limited to 20 months of age.

In conclusion, brief postnatal ACEi in SFK sheep delayed the onset of albuminuria by at least 8 months. However, the reno-protective benefits of early ACEi in SFK diminished with age. GFR was lower in SFK+ACEi animals compared with SFK 18 months after the withdrawal of treatment. But whether this lower GFR will delay or accelerate loss of kidney function requires further investigation. At 20 months of age, in response to AA+D although GFR and RBF increased in all groups, the increase in RBF was greater in the SFK+ACEi group than in SFK. This greater vasodilation of SFK vasculature may indicate some long-lasting kidney functional effects of brief ACEi early in life. Modulation of the window or duration of ACEi needs to be investigated to determine if sustained long term effects can be achieved to benefit children with SFK.

## Clinical perspectives

- Onset of hypertension and kidney disease in children born with a solitary functioning kidney (SFK) can occur early in life as a result of hyperfiltration mediated injury. A brief period of angiotensin-converting enzyme inhibition (ACEi) early in life in sheep with SFK is reno protective at 8 months of age, but the long-term consequences of this treatment are unknown.
- Reno-protective benefits of brief early ACEi in SFK waned with age with albuminuria similar to untreated levels by 14 months of age. But some residual changes in kidney hemodynamics remained at 20 months of age.
- Brief postnatal ACEi alters the trajectory of kidney disease in SFK. A better understanding of the adaptation of kidney function due to this treatment and alterations in the duration of ACE inhibition to prolong benefits is required to improve long-term outcomes in children with SFK.

## Data Availability

The data underlying this article will be shared on reasonable request to the corresponding author.

## Open Access

Open access for this article was enabled by the participation of Monash University in an all-inclusive *Read & Publish* agreement with Portland Press and the Biochemical Society under a transformative agreement with CAUL.

## Abbreviations

ACEi: angiotensin-converting enzyme inhibition
BP: blood pressure
CKD: chronic kidney disease
GFR: glomerular filtration rate
HR: heart rate
MAP: mean arterial pressure
NO: nitric oxide
PRA: plasma renin activity
RBF: renal blood flow
RFR: renal functional reserve
RVR: renal vascular resistance
SFK: solitary functioning kidney
